# Mice Null for Calsequestrin 1 Exhibit Deficits in Functional Performance and Sarcoplasmic Reticulum Calcium Handling

**DOI:** 10.1371/journal.pone.0027036

**Published:** 2011-12-02

**Authors:** Rotimi O. Olojo, Andrew P. Ziman, Erick O. Hernández-Ochoa, Paul D. Allen, Martin F. Schneider, Christopher W. Ward

**Affiliations:** 1 Department of Biochemistry, University of Maryland School of Medicine, Baltimore, Maryland, United States of America; 2 Department of Physiology, University of Maryland School of Medicine, Baltimore, Maryland, United States of America; 3 Department of Anesthesia, Perioperative and Pain Medicine, Brigham and Women's Hospital, Boston, Massachusetts, United States of America; 4 University of Maryland School of Nursing, Baltimore, Maryland, United States of America; University of California Merced, United States of America

## Abstract

In skeletal muscle, the release of calcium (Ca^2+^) by ryanodine sensitive sarcoplasmic reticulum (SR) Ca^2+^ release channels (i.e., ryanodine receptors; RyR1s) is the primary determinant of contractile filament activation. Much attention has been focused on calsequestrin (CASQ1) and its role in SR Ca^2+^ buffering as well as its potential for modulating RyR1, the L-type Ca^2+^ channel (dihydropyridine receptor, DHPR) and other sarcolemmal channels through sensing luminal [Ca^2+^]. The genetic ablation of CASQ1 expression results in significant alterations in SR Ca^2+^ content and SR Ca^2+^ release especially during prolonged activation. While these findings predict a significant loss-of-function phenotype *in vivo*, little information on functional status of CASQ1 null mice is available. We examined fast muscle *in vivo* and *in vitro* and identified significant deficits in functional performance that indicate an inability to sustain contractile activation. In single CASQ1 null skeletal myofibers we demonstrate a decrease in voltage dependent RyR Ca^2+^ release with single action potentials and a collapse of the Ca^2+^ release with repetitive trains. Under voltage clamp, SR Ca^2+^ release flux and total SR Ca^2+^ release are significantly reduced in CASQ1 null myofibers. The decrease in peak Ca^2+^ release flux appears to be solely due to elimination of the slowly decaying component of SR Ca^2+^ release, whereas the rapidly decaying component of SR Ca^2+^ release is not altered in either amplitude or time course in CASQ1 null fibers. Finally, intra-SR [Ca^2+^] during ligand and voltage activation of RyR1 revealed a significant decrease in the SR[Ca^2+^]_free_ in intact CASQ1 null fibers and a increase in the release and uptake kinetics consistent with a depletion of intra-SR Ca^2+^ buffering capacity. Taken together we have revealed that the genetic ablation of CASQ1 expression results in significant functional deficits consistent with a decrease in the slowly decaying component of SR Ca^2+^ release.

## Introduction

Specialized systems have evolved for the robust and rapid release of calcium (Ca^2+^) ions from the sarcoplasmic reticulum (SR) in skeletal and cardiac muscle. During skeletal muscle activation, a single action potential initiates a release of ∼200 µM of Ca^2+^ (per liter of fiber volume) from the SR which causes a “twitch” contraction. As the SR [Ca^2+^] in fast muscle is at approximately 30–40% of maximal capacity at rest, several groups have estimated twitch release to be between 10–18% of the total releasable SR Ca^2+^ in fast twitch muscle from frog or mouse [1], [2], [3], [4]. However, repetitive trains of action potentials are a hallmark of skeletal muscle activation. These contractions are sustained by the reserve of releasable Ca^2+^ in the basal state and a rapid restoration of SR Ca^2+^ content due to increased sarco-endoplasmic reticulum ATPase activity (SERCA) and dynamic refilling of the SR through sarcolemmal Ca^2+^ influx (i.e., excitation-coupled calcium entry; ECCE [5], [6], [7]) during repetitive stimuli. Recent work to elucidate the control of this dynamic regulation of SR Ca^2+^ has focused on calsequestrin (CASQ) and its binding partners in the SR membrane as potential modulators of the RyR1, the DHPR and other sarcolemmal channels through sensing luminal [Ca^2+^] [8], [9], [10].

Calsequestrins are acidic, high capacity Ca^2+^ binding proteins in the SR. Two isoforms of mammalian calsequestrin [11], [12] have been identified and characterized: a skeletal muscle and a cardiac muscle isoform, or CASQ1 and CASQ2 respectively. CASQ2 is the only isoform expressed in the heart. Whereas both CASQ1 and 2 are expressed in various skeletal muscles, fast muscle only contains CASQ1 while slow muscle contains both isoforms [13], [14]. CASQ1 facilitates the accumulation of Ca^2+^ within the SR. The molecular basis for the tremendous binding capacity of CASQ1 is in its behavior of forming polymer aggregates at high [Ca^2+^]. While the CASQ1 polymer networks facilitate an accumulation of Ca^2+^ close to the RyR, it is also clear that the CASQ1 polymer arrangement facilitates the rapid off-loading of Ca^2+^ during RyR channel opening [3]. During relaxation, CASQ1 functions as a buffer of Ca^2+^ in the SR lumen keeping the free concentration relatively low and thus allowing more efficient inward transport by the SERCA pumps [15], [16], [17], [18]. This is particularly important in fast-twitch fibers where the amount of Ca^2+^ released and taken up is much greater than in slow fibers [19]. The higher concentration of CASQ1 in fast fibers [20], [21] reflects its presumed important role in increasing the Ca^2+^ content within the SR during repetitive contraction [22].

Genetic ablation of CASQ expression has proved to be an important tool in elucidating the role of CASQ in cardiac and skeletal muscles. Initial findings support the conclusion that CASQ is NOT essential for EC coupling as ablation of CASQ1 expression in *C. elegans* [23], or ablation of CASQ1 [24] and both CASQ1 and 2 [25] expression in mice were not lethal. In fact null animals thrived and reproduced normally. Subsequent studies in CASQ1 null mice [26] demonstrated little functional alteration of isolated fast skeletal muscle and only a mild reduction in global Ca^2+^ signaling with single twitch stimulation. Since this report, a detailed examination with prolonged depolarization in CASQ1 null myofibers revealed reduced peak and total SR Ca^2+^ release flux [27]. Most recently examination of both cytosolic transients and intra-SR depletion kinetics with prolonged depolarization in the CASQ1 null and CASQ1/2 double null mice, revealed a decrease in the ability to sustain SR release flux [28] in null fibers. However despite uncovering significant deficits at the level of the SR in CASQ1 null mice these individual studies have provided few functional correlates as a result of these deficits.

In this investigation we examined functional measures of muscle performance *in vivo* and *in vitro*, measures of voltage dependent SR Ca^2+^ release and SR Ca^2+^ release flux, as well as measures of SR [Ca^2+^] and its depletion during stimulation. We demonstrate that genetic ablation of CASQ*1* expression results in significant deficits in functional performance *in vivo*. Furthermore, while *in vitro* analysis of muscle contractility revealed minimal alterations in maximal contractile activation, dramatic alterations in the ability to sustain contractile function were seen with prolonged trains of physiologic stimuli. Consistent with a steady reduction in the amount of Ca^2+^ available to allow contractile filament activation, we identified a collapse of SR Ca^2+^ release in Indo1 loaded CASQ1 null myofibers as well as a decrease in SR Ca^2+^ release flux and total SR Ca^2+^ release in voltage clamped CASQ1 null myofibers. Finally, CASQ1 null myofibers had a significant reduction in total releasable SR [Ca^2+^] and altered kinetics of release and uptake consistent with the lack of a significant SR Ca^2+^ buffer. Taken together we have demonstrated that the genetic ablation of CASQ1 expression results in significant deficits in SR Ca^2+^ storage and release which in-turn contribute to significant deficits in contractile activation and whole animal functional performance.

## Results

### CASQ1 null mice exhibit decrements in gross neuromuscular performance

**Figure 1:**
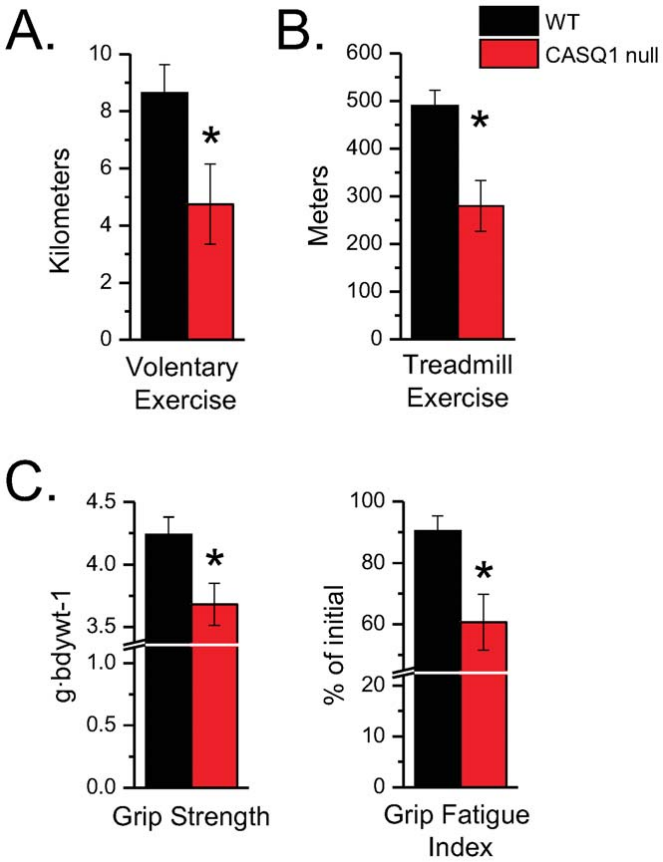
Neuromuscular phenotyping *in vivo*. **A.** Voluntary exercise capacity: Mice (CASQ1 null; n = 6) and WT; n = 6) were singly housed in a cage with a mouse running wheel (Lafayette Instruments, Lafayette, IN) in a 12/12 hour light/dark animal facility with *ad libitum* access to food and water. The number of wheel revolutions were computer analyzed per 24 hour period and data was converted to mean number of meters per day and reported as descriptive data. When compared to the WT group (8.64±0.99 km), the CASQ1 null animals (4.75±1.41 km) exhibited a significant deficit in total daily running distance (t-test, p<0.05) **B.** Treadmill exercise capacity (i.e., non-voluntary) was evaluated in previously sedentary CASQ1 null (n = 6) and WT (n = 6). Following an a 3 day acclimation period (see methods), mice were exercised at 10 meters/minute for the first 10 min then ramped to 20 m/min over 1 min and held there until volitional exhaustion. Volitional exhaustion was assessed by a single rater, blinded to mouse genotype, who determined the time point at which the mouse was unable to maintain treadmill pace after two successive startles. After the trial, total time was converted to distance covered. Exercise naïve WT mice performed significantly better (490±80 m; n = 6) than age matched CASQ1 null mice (280±130 km; n = 6). **C.** Forepaw grip strength was determined on a commercial rodent grip strength meter (Columbus Inst., USA) as described (see methods). CASQ1 null mice (n = 8) exhibited a significant deficit in grip strength (C; left panel) compared to WT (n = 8) mice when normalized to bodyweight (3.68±0.17 vs. 4.24±0.14 g·bdwt^−1^; t-test, p<0.05). The body weight of the WT mice was approximately 18% larger than those of the CASQ1 null animals (34.58±0.26 g vs. 28.14±1.36; t-test, p<0.05). The decrement in force between the peak value and the value of the 7^th^ trial was evaluated as an index of fatigue (C; right panel) and expressed as % of initial.CASQ1 null animals exhibited more fatigue than did their WT counterparts.

Voluntary exercise capacity has recently been used as a sensitive measure of deficits caused by the deletion of putative EC coupling proteins [29]. Here we provided exercise naïve CASQ1 null (n = 6) and WT (n = 6) mice free access to computerized running wheels for 14 days. The mean 24 hour running distance from the final three days was taken as the voluntary exercise capacity. CASQ1 null animals exhibited a significant deficit in total daily running distance compared to the WT group (Fig. 1A). Likewise, treadmill exercise capacity (i.e., forced exercise; Fig. 1B) was significantly reduced in CASQ1 nulls compared to age matched WT mice.

Forepaw grip strength, a sensitive assay used to detect dysfunction in several murine models of motor neuron [30], [31], [32] and muscle diseases [33], was used as an indirect measure of gross neuromuscular performance. The peak force in each of 7 successive trials was determined and the 2 highest values were averaged and normalized to bodyweight. As seen in Fig. 1C (left), CASQ1 null mice (n = 8) exhibited a significant deficit in grip strength normalized to body weight compared to WT mice (n = 8). As seen previously [24], the body weight of the WT mice was approximately 18% greater than that of the CASQ1 null animals. The relative drop in the peak grip strength over the 7 trials was assessed as the index of grip strength fatigue. As seen in Fig. 1C (right), CASQ1 null animal exhibited more fatigue over the 7 successive trials than did their WT counterparts.

### Assays of muscle contractility reveal significant deficits in CASQ1 null mice

**Figure 2:**
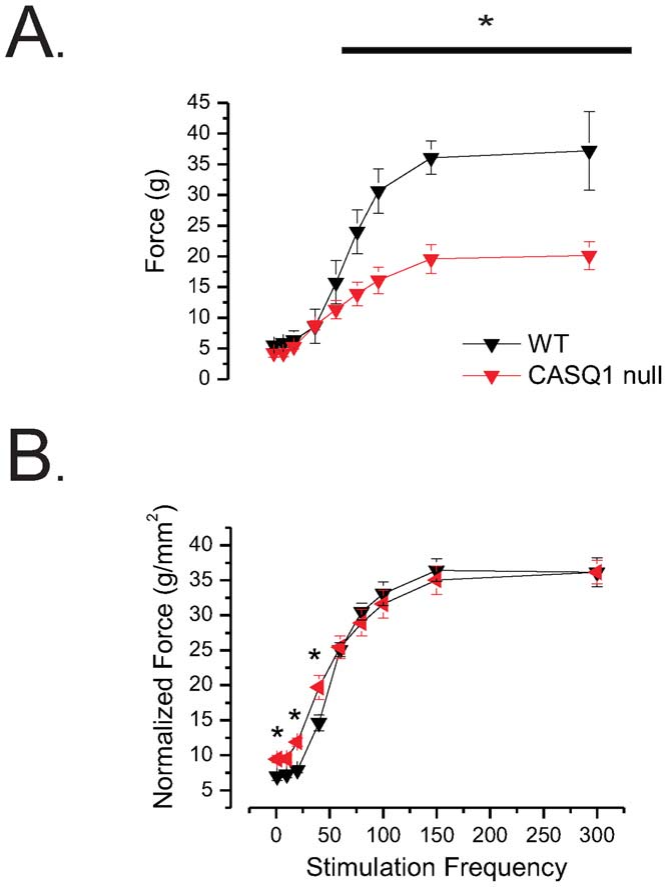
EDL Force vs. stimulation frequency relationship assayed *in vitro*. Trains of pulses (0.2 msec.) delivered at 1, 30, 50, 80, 100,150 and 300 Hz. **A.** The absolute isometric force vs. stimulation frequency relationship between WT and CASQ1 null EDLs revealed significantly reduced tension in the CASQ1 nulls between 40–300 Hz. However, EDL muscles from the CASQ1 null mice (7.58±0.43 mg; n = 8) were approximately 29% smaller in mass than those from the WT age matched controls (10.73±0.71 mg; n = 8, t-test, p<0.05). **B.** Following CSA normalization the force vs. stimulation frequency relationship revealed small but significant differences in the amplitudes of low frequency trains (1 and 20 Hz and 40 Hz) while no differences were seen at any other frequency (ANOVA).

To evaluate the muscle specific contribution to the deficits seen in the neuromuscular function assays, we examined muscle contractility *in vitro* in explanted whole EDL muscles surgically excised from age matched WT and CASQ1 null mice as we have previously described [34], [35]. Each muscle's tendons were affixed with ligatures between a fixed post and a tension transducer (isometric mode) in a bath and the muscle superfused with oxygenated physiologic saline. Isometric tension was evaluated with trains of pulses delivered at 1, 30, 50, 80, 100,150 and 300 Hz. Figure 2A displays the peak isometric force vs. stimulation frequency relationship between EDLs from WT (n = 8) and CASQ1 null (n = 8) mice. While a large decrease in absolute peak force output was apparent in CASQ1 null muscles, these results were likely influenced by the significant differences in the muscle wet weight as EDL muscles from the CASQ1 null mice were approximately 29% smaller in mass than those from the WT age matched controls. To allow direct comparison of specific force generation between genotypes, the peak isometric tension produced was normalized to muscle cross sectional area (CSA) [36] (see methods).

Following CSA normalization there were small but significant differences in the amplitudes of the force vs. stimulation frequency relationship (Fig. 2B) at low frequency trains (1 and 20 Hz and 40 Hz) but there were no differences were seen at any other frequency, indicating that the majority of the loss in absolute force in un-normalized data was due to decreased muscle mass. Kinetic analysis revealed a significant decrease in the time-to-peak of the twitch in the CASQ1 null EDLs at the 1 Hz stimulus frequency without any difference in the maximal rate of rise (+dP/dT). However, examination of the maximal tetanic transient (300 Hz) revealed that both time-to-peak and +dP/dT were faster during tetanic contractions in CASQ1 null EDLs compared to WT (Table 1).

**Table 1:** Kinetic parameters of EDL contractility *in vitro*.

	WT		CASQ1 null	
**EDL Weight(mg)**	10.73±0.71∧		7.58±0.43∧	

	Twitch(1 Hz)	Tetanic (300 Hz)	Twitch(1 Hz)	Tetanic (300 Hz)
**Force (g)**	5.72±0.45	36.66±2.93^&^	5.58±0.96	19.18±2.1^&^
**Max dp/dt (msec)**	3.75±1.8	8.0±2.5^#^	4.0±1.4	14.6±3.9^#^
**Time topeak(s)**	0.101±0.012 ^*^	0.093±0.008^∧^	0.045±0.018^*^	0.065±0.013^∧^

Like symbols indicate significance p<0.05.

**Figure 3:**
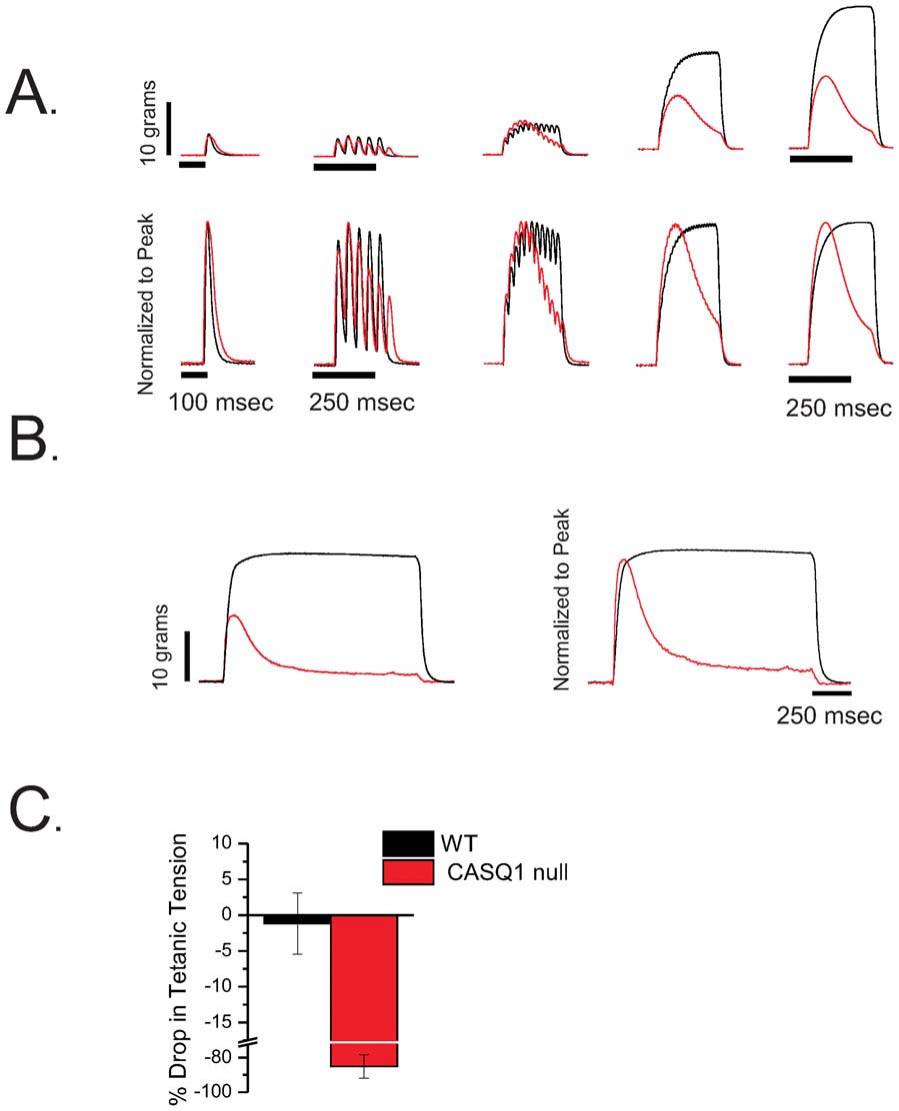
Representative raw force records from WT and CASQ1 null mice. **A** Raw records (top) and same records normalized to peak tension (**A**, bottom) from the EDL force vs. frequency experiment (WT = black, CASQ1 null = red) with 500 msec trains of pulses of 1, 20, 40, 80, 300 Hz. (see Fig. 2A for aggregated data of maximal tension). CASQ1 null EDL muscles exhibited a frequency dependent inability to sustain force during the pulse train. Note that significant difference in maximal force generation normalized when the differences in muscle mass were accounted for (see Fig. 2B.). **B.** Representative normalized force traces from a WT and CASQ1 null muscle in which a 1500 msec train of pulses was delivered at 100 Hz. **C.** In 4 EDL muscles from each genotype, we demonstrated that while WT muscle showed little change in tension over the 1500 msec, developed tetanic tension in the CASQ1 null muscles fell to 85.2±6.7% (t-test, p<0.05) of the peak value.

Despite the similarity of the peak of normalized force production by the WT and CASQ1 null EDL muscles, visual inspection of the raw force records revealed dramatic differences in the time course between the two genotypes. As seen in representative raw records from the force vs. frequency experiment, (1, 20, 40, 80, 300 Hz; Fig. 3A, top) CASQ1 null EDLs were unable to sustain peak force during trains of action potentials, and exhibited a significant reduction of tension during the train. The temporal relationship of this decline is more evident when these traces are normalized to the peak of the transient (Fig. 3A, bottom). The inability of CASQ1 null muscles to maintain their isometric force was even more evident when we increased the duration of the pulse train from 300 to 1500 msec. Fig. 3B displays a representative set of raw traces from a WT and CASQ1 null muscles during 1500 msec train of 100 Hz pulses. In this experiment, it is clear that the fade in isometric tension in the CASQ1 null muscle reached a nadir of 85.2±6.7% from the peak value by 800 msec while WT muscle showed little change in tension over the 1500 msec (Fig. 3C).

Taken together, these results suggest that while the EDL muscle from CASQ1 null animals has significant deficits in total force output, when normalized to muscle cross sectional area force output is only mildly altered (Fig. 2). Furthermore, while CASQ1 null muscle was able to activate the contractile apparatus in similar fashion as the WT, the CASQ1 null EDLs had a dramatic fade in the temporal evolution of the force transient during a 1500 msec pulse train.

### FDB myofibers from CASQ1 null mice exhibit significant deficits in electrically evoked Ca^2+^ transients

As Ca^2+^ release from the sarcoplasmic reticulum (SR) is a positive predictor of myofiber contractile force generation [37], we next asked whether alterations in Ca^2+^ release from the SR, contributed to the significant ‘fade’ in force phenotype seen in muscles from CASQ1 null mice.

### Action potential-evoked Indo1 Ca^2+^ transients are altered in CASQ1 null muscle fibers

**Figure 4:**
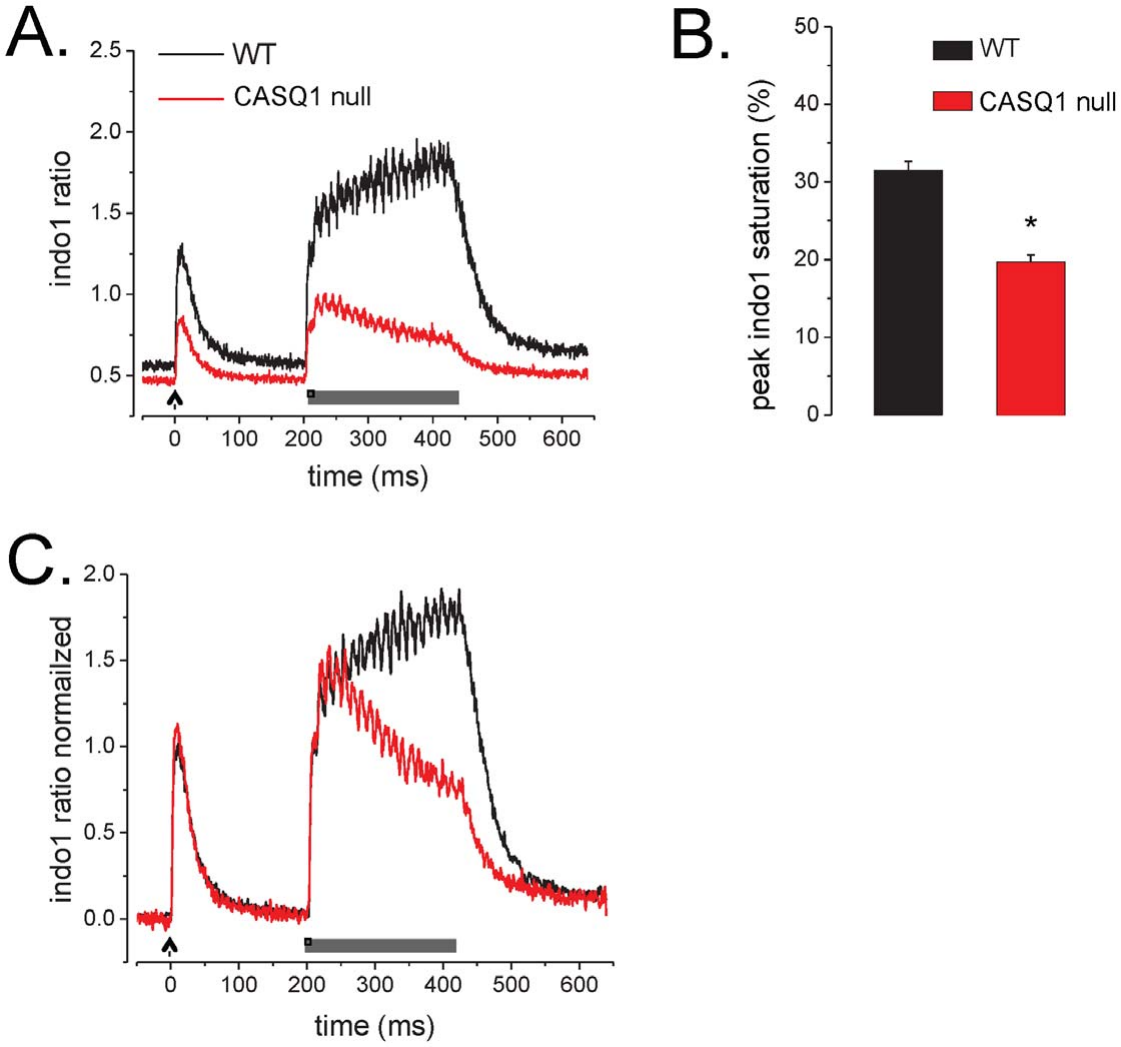
CASQ1 null muscle fibers exhibit a decreased peak amplitude of action potential-evoked indo-1 Ca^2+^ transient. **A.** Average indo-1 transient responses to a single AP stimuli followed by a 250 msec. 100 Hz train of APs from WT (*black trace*; n = 13) and CASQ1 null (red *trace*; n = 21) fibers. Indo-1 responses show reduced amplitude of Ca^2+^ transients after single and tetanic stimulation in CASQ1 null fibers. **B.** Bar plot summarizing the peak indo 1 transient after correcting for indo-1 saturation. Significant differences in peak Indo-1 were found in CASQ1 null fibers (WT = 1.33±0.04, CASQ1 null = 0.83±0.03, p<0.05,, *p*<0.05). **C.** Traces from *A* normalized to initial transient amplitude to demonstrate lack of summation of the Ca^2+^ transient during repetitive stimulation of CASQ1 null fibers when compared to WT counterpart (last/first ratio WT = 1.91±0.02, n = 13; CASQ1 null = 0.81±0.04, n = 21; Mann-Whitney rank-sum test, p<0.05).

First, we sought to evaluate action-potential evoked Ca^2+^ transients and Ca^2+^ homeostasis in single, intact WT and CASQ1 null muscle fibers stimulated by single or repetitive field stimulation. FDB fibers from 5–7 week old mice were loaded with the ratiometric Ca^2+^ indicator Indo1-AM and after dye conversion stimulated with field electrodes as previously described [38]. Fig. 4A illustrates average responses from 13 control and 21 CASQ1 null fibers stimulated by a single (1 ms; 14 V) field stimulus followed by a train (240 ms) of 1 ms/14 V pulses at 100 Hz. There was a significant reduction in the cytosolic Ca^2+^ transient in CASQ1 null fibers in response to a single AP (Fig. 4B, right bar plot). This difference was even more striking during repetitive stimulation where compared to WT counterparts there was a rapid collapse of the Ca^2+^ transient in the CASQ1 null fibers (Fig. 4A). Consistent with single stimulation, the first indo1 Ca^2+^ transient elicited during a train was reduced in CASQ1 null (Fig. 4A; red trace) compared to WT fibers (Fig. 4A; black trace) and then unlike WT fibers the CASQ1 null fibers were unable to maintain the summation of calcium signal during repetitive stimulation (Fig. 4A). Figure 4C shows the average WT and CASQ1 null indo1 ratios normalized to the initial peak of the last train. Both WT and CASQ1 null fibers showed an initial increase in the peak amplitude during the first 100 ms of 100 Hz train. However, while the subsequent transients during the train in WT fibers continued to rise, in CASQ1 null fibers the relative amplitude of the Ca^2+^ transients during the train declined significantly (Fig. 4C).

### SR Ca^2+^ release flux is suppressed in CASQ1 null myofibers

**Figure 5:**
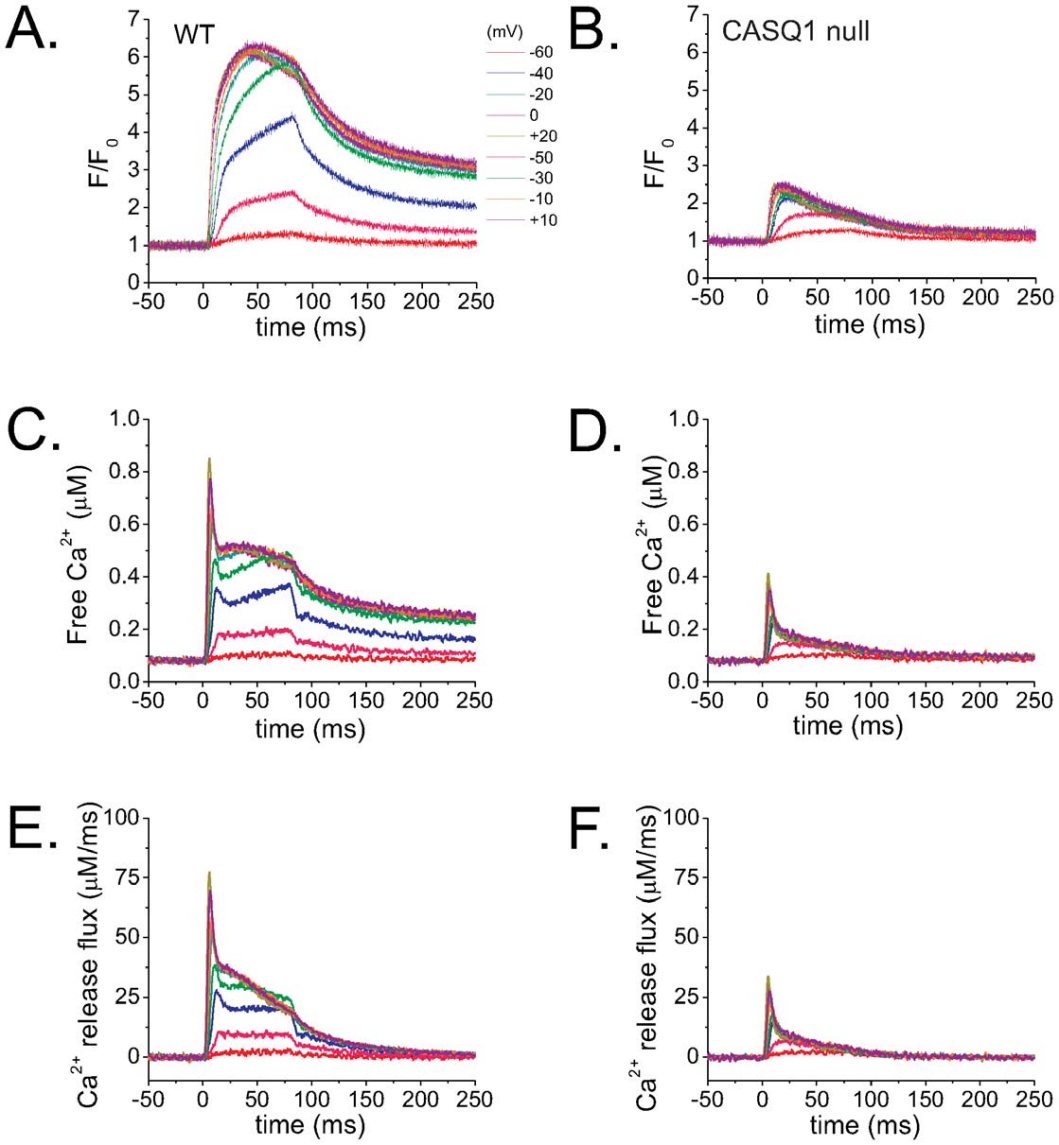
Fluo-4 fluorescence transients are altered in CASQ1 null FDB myofibers. Free [Ca^2+^] and Ca^2+^ release flux from WT (left) and CASQ1 null fibers (right). Average (n = 4) time course of fluo-4 F/F_0_ records expanded in time elicited at different voltages are displayed for WT fibers (**A**) and for CASQ1 null fibers (**B**). (**C**) and (**D**) are free [Ca^2+^] waveforms derived from *A* and *B* while (**E**) and (**F**) are Ca^2+^ release flux calculated from *C* and *D*. Comparison of the two sets of data show significant suppression of the amplitude of F/F_0_, free Ca^2+^ and peak and maintained Ca^2+^ release flux in the CASQ1 null fibers.

Fig. 5 shows averaged time course of fluo-4 Ca^2+^ transients elicited by 80 ms step depolarization to a range of voltages and expressed as F/F_0_ obtained from WT (Fig. 5A, N = 4) and CASQ1 null fibers (Fig. 5B, N = 4). The pulse protocol utilized both an initial and final voltage pulse to −20 mV and a series of intervening pulses to −60 mV to +20 mV at 20 mV step increments, followed by another sequence of pulses ranging from +10 to −50 mV, decreasing the amplitude in 20 mV increments, with a reference pulse to −20 mV at the start, middle and end of the sequence, as shown in Fig. 5. A correction for possible changes in the fibers resting fluorescence over the course of each experiment was applied by normalizing all records in the second sequence by the ratio of the ΔF/F_0_ values, obtained by dividing ΔF/F_0_ from the middle pulse from the first sequence to that in the second sequence in order to arrive at the corrected F/F_0_ records (Fig. 5A and 5B). These records were used to calculate the free cytosolic Ca^2+^ concentration (free [Ca^2+^]) by using Eq. 3 (see methods).

**Figure 6:**
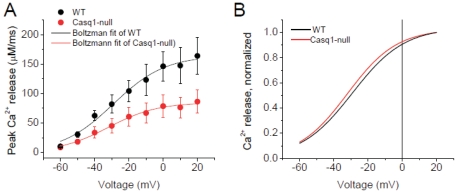
Peak Ca^2+^ release flux vs. voltage relationship in WT and CASQ1 null myofibers. **A.** Peak Ca^2+^ release flux was plotted as a function of voltage in CASQ1 null and WT fibers. Data was fitted to a single Boltzmann function with the following parameters: *R_max_*, *V_half_* and *k* parameters were 32.6 µMms^−1^, −33.4 mV and 15.2 mV and 73.6 µMms^−1^, −30.9 mV and 12.3 mV for CASQ1 null and WT fibers respectively. **B.** Peak release flux *vs.* voltage normalized to the R_max_ allows for better estimation of the effect the absence of CASQ1 on voltage dependence and on the steepness of Ca^2+^ release. Non-significant differences in the voltage-dependence parameters of Ca^2+^ release from CASQ1 nulls were found.

The estimation of the Ca^2+^ release flux in each fiber was then calculated from its free Ca^2+^ waveform. For each fiber, the rate of Ca^2+^ release from the inside of the SR into the cytosol was calculated using Eq. 5. Figs. 5C and 5E represent the average of the records of cytosolic free [Ca^2+^] and the Ca^2+^ release flux from 4 WT fibers, while corresponding data on the right (Fig. 5D and 5F) are the averages from 4 CASQ1 null fibers. The absence of CASQ1 greatly suppressed the average fluo-4 signal at each voltage, as well as the calculated free Ca^2+^, the magnitude of the initial peak and the later more sustained component of Ca^2+^ release for all pulses. Using data obtained from each of the fibers in Fig. 5E and 5F, values of the peak release rates were calculated and plotted as a function of voltage as illustrated in Fig. 6A. The data were fitted to a single Boltzmann function described by the following equation [38]:
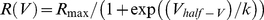
(6)where *R_max_* is the maximum release rate, *V_half_* defines the potential when R = 0.5 *R_max_*, and 1/*k* is a measure of the steepness of the R-V relationship. Average *R_max_*, *V_half_* and *k* parameters were 32.6 µMms^−1^, −33.4 mV and 15.2 mV and 73.6 µMms^−1^, −30.9 mV and 12.3 mV for CASQ1 null and WT fibers respectively. This analysis reveals a 55% decrease (N = 4) in the maximum peak Ca^2+^ release in the CASQ1 null fibers compared to WT. Fig. 6B plots the R-V curves normalized to *R_max_*, allowing a better visualization of effect of ablation of CASQ1 expression on the voltage dependence and steepness of the Ca^2+^ release. The *V_half_* values shown here indicate a non-significant shift of 3 mV in the voltage-dependence of Ca^2+^ release in CASQ1 null animals (Fig. 6A and B).

**Figure 7:**
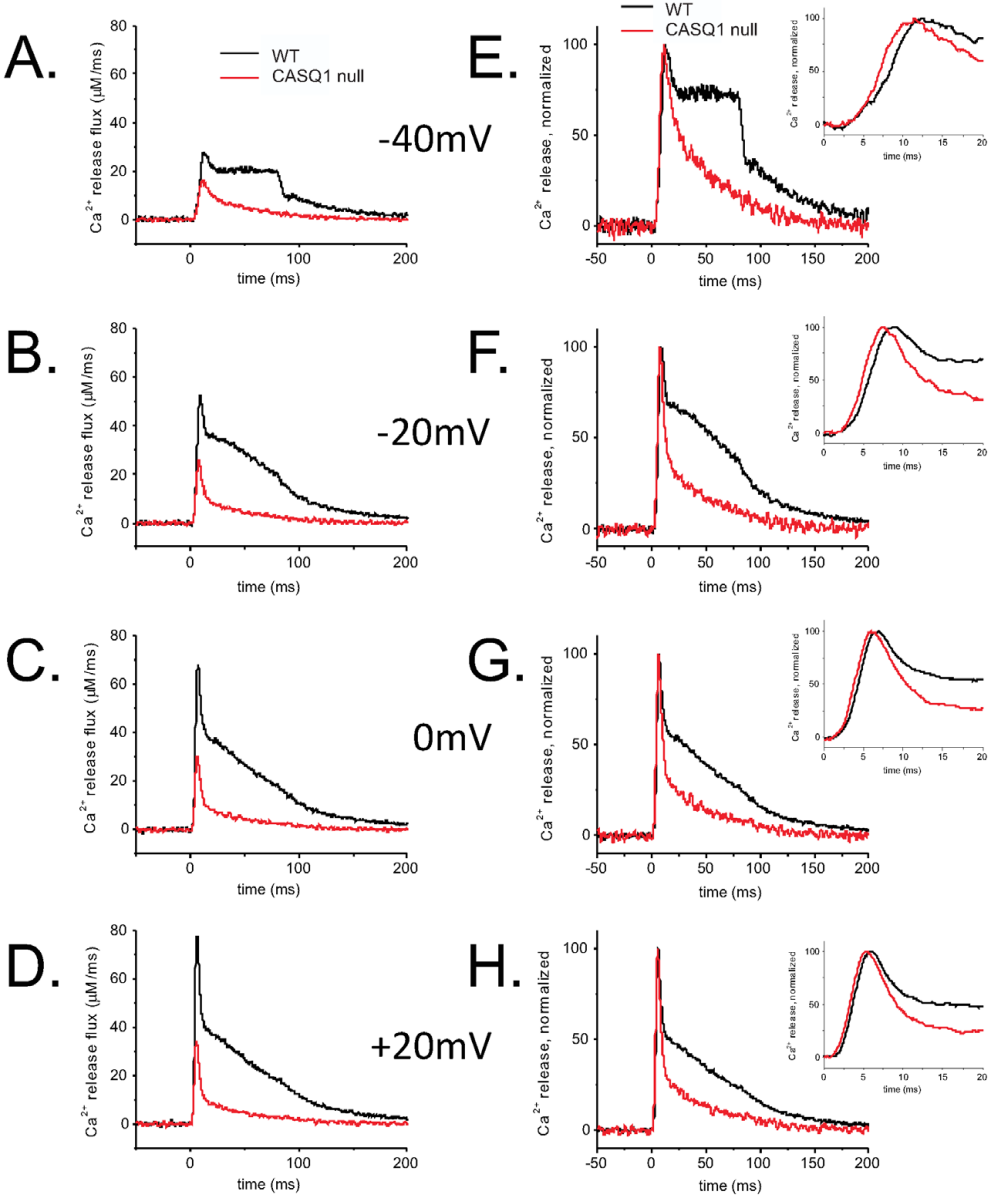
The kinetics of the Ca^2+^ release time courses differ between CASQ1 null and WT myofibers. Superimposed average Ca^2+^ release time courses at −40 mV (**A**), −20 mV (**B**), 0 mV (**C**) and +20 mV (**D**) voltage steps from WT (black traces) and CASQ1 null fibers (red traces). (**E**–**H**) Time course of the normalized Ca^2+^ release flux in the WT and CASQ1 null fibers derived from panels *A–D* to show effects of CASQ1 elimination on the kinetics of Ca^2+^ release. *Insets* show zoom-in versions of the raising phase of the rate of Ca^2+^ release. The kinetics of the peak formation appear unchanged from the WT fibers to the CASQ1 null fibers however the slow component is virtually eliminated, suggesting a rapid decline of the SR Ca^2+^ content in the absence of CASQ1.

Figures 7A–D present superimposed Ca^2+^ release flux records in WT and CASQ1 null fibers for 4 different voltages. The figures show a very significant reduction in the peak values of the Ca^2+^ release flux at voltages that elicited Ca^2+^ release i.e., −40 mV, −20 mV, 0 mV, and +20 mV. The peak Ca^2+^ release flux of the CASQ1 null fibers were more strongly suppressed relative to WT fibers at higher depolarizations. In addition, the total amount of Ca^2+^ released from the SR at each voltage, which corresponds to the time integral or area of the release flux records (data not shown) is markedly decreased in the CASQ1 null fibers, consistent with a greatly reduced total SR Ca^2+^ content.

In WT fibers, each of the Ca^2+^ release waveforms for larger depolarizations (e.g., 0, +20 mV) is characterized by a pronounced early peak that occurs within 10 ms of the application of the pulse(Fig. 7C–D; black traces). This is then followed by an initial rapid decline and then a much slower decline, with a temporal profile that is believed to reflect an early partial inactivation of RyR1 followed by a time dependent decline of the SR Ca^2+^ content and/or a partial decline in SR Ca^2+^ permeability [39], [40], [41]. In this interpretation, the early peak represents the maximum amplitude of the sum of the amplitudes of the rapidly decaying and the slowly decaying component of Ca^2+^ release at each voltage. The amplitude of the shoulder just after completion of the rapid phase of decline in the release flux records from the WT fibers represents the initial magnitude of the slowly decaying phase of release flux. In considering the release records from this perspective, it appears that the slowly decaying phase of decay of release flux is almost totally abolished in the CASQ1 null fibers. In contrast, the rapidly decaying component is hardly altered. In order to compare the kinetic properties of the Ca^2+^ release flux in fibers from WT and CASQ1 null mice, Ca^2+^ release flux time courses for CASQ1 null and control (WT) fibers were plotted together and normalized to the peak values at voltages that elicited Ca^2+^ release (i.e. −40 mV, −20 mV, 0 mV, and +20 mV; Fig. 7E–H). The kinetics of the peak formation and time to peak appear no different in WT and CASQ1 null fibers at all voltages examined as illustrated by the time-expanded insets in Figures 7E–H. However, the rate of decay of the total release records in CASQ1 null fibers is similar in time course to the rate of decay of the fast component in the WT fibers, so much so that the slow component is virtually eliminated (Fig. 7A–H).

**Figure 8:**
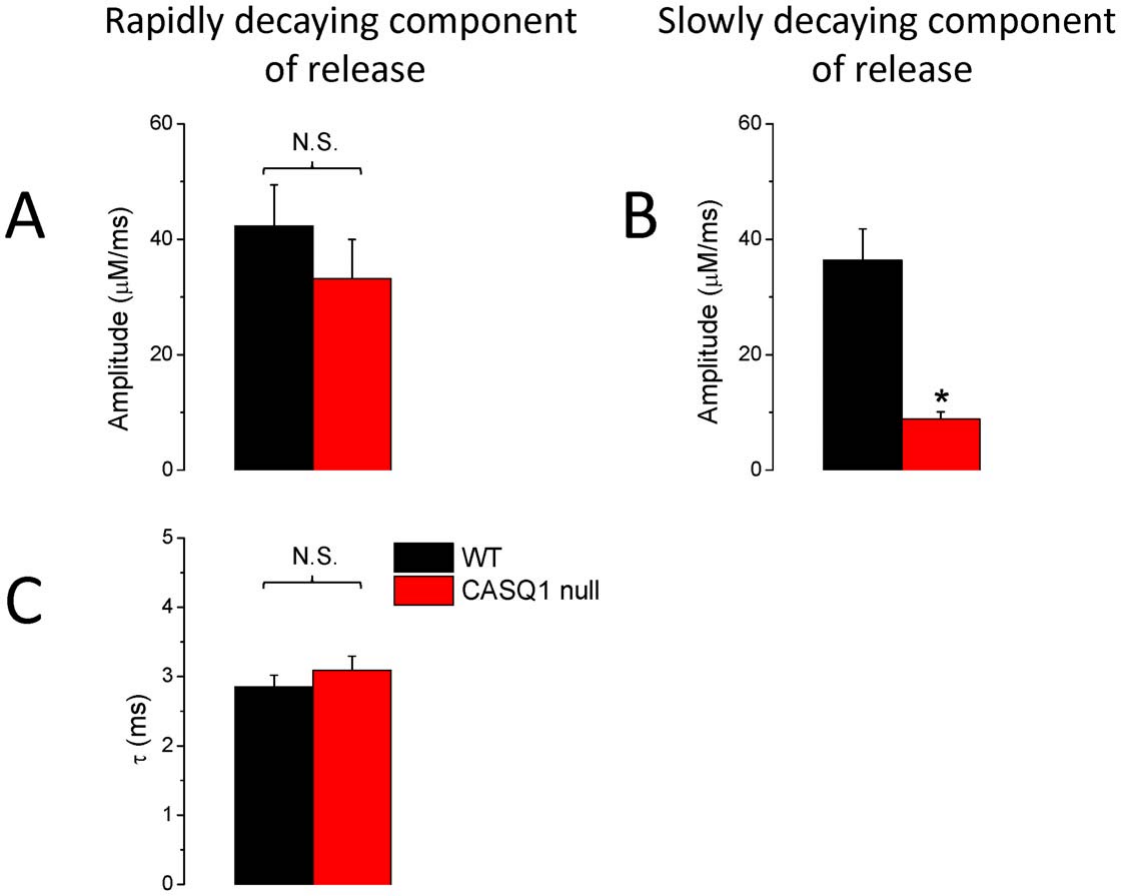
Kinetics of the rapidly decaying component of the release flux at 20 mV in WT and CASQ1 null fibers. A decaying exponential fit to the Ca^2+^ release flux at 20 mV was used to determine the amplitude and time constant of the rapidly decaying component of the release flux in both WT and CASQ1 null fibers. The offset of the exponential fit corresponds to an estimate the amplitude of the slow decaying component of the release. **A** and **B** independently show comparison of the amplitudes of the fast and of the slow decaying component in both WT and CASQ1 null fibers respectively (n = 7). **B** shows that the amplitude of the slowly decaying component of release in CASQ1 null fibers was significantly reduced when compared to WT counterparts (* denotes p<0.05) while no significant changes occurred in the amplitude and the decay time constant of the rapidly decaying component of release in CASQ1 null fibers.

Figure 8 quantitatively compares the properties of the 2 phases of decay of Ca^2+^ release in WT and CASQ1 null fibers. The amplitude and time constant of decay of the rapidly decaying component were obtained from a single exponential fit to the decay of the rapid component from the beginning of its decay to 20 ms after the start of the pulse for pulses to +20 mV. The amplitude of the slow phase was taken as the constant in the same single exponential plus constant fits. There was no significant difference in the amplitude or decay time constant of the rapidly decaying component of release. In contrast, the mean amplitude of the slowly decaying component of release was significantly reduced in the null fibers to 24% of the mean value for the control fibers (p<0.05), further supporting the selective suppression of the slowly decaying component of release in CASQ1 null fibers.

**Figure 9:**
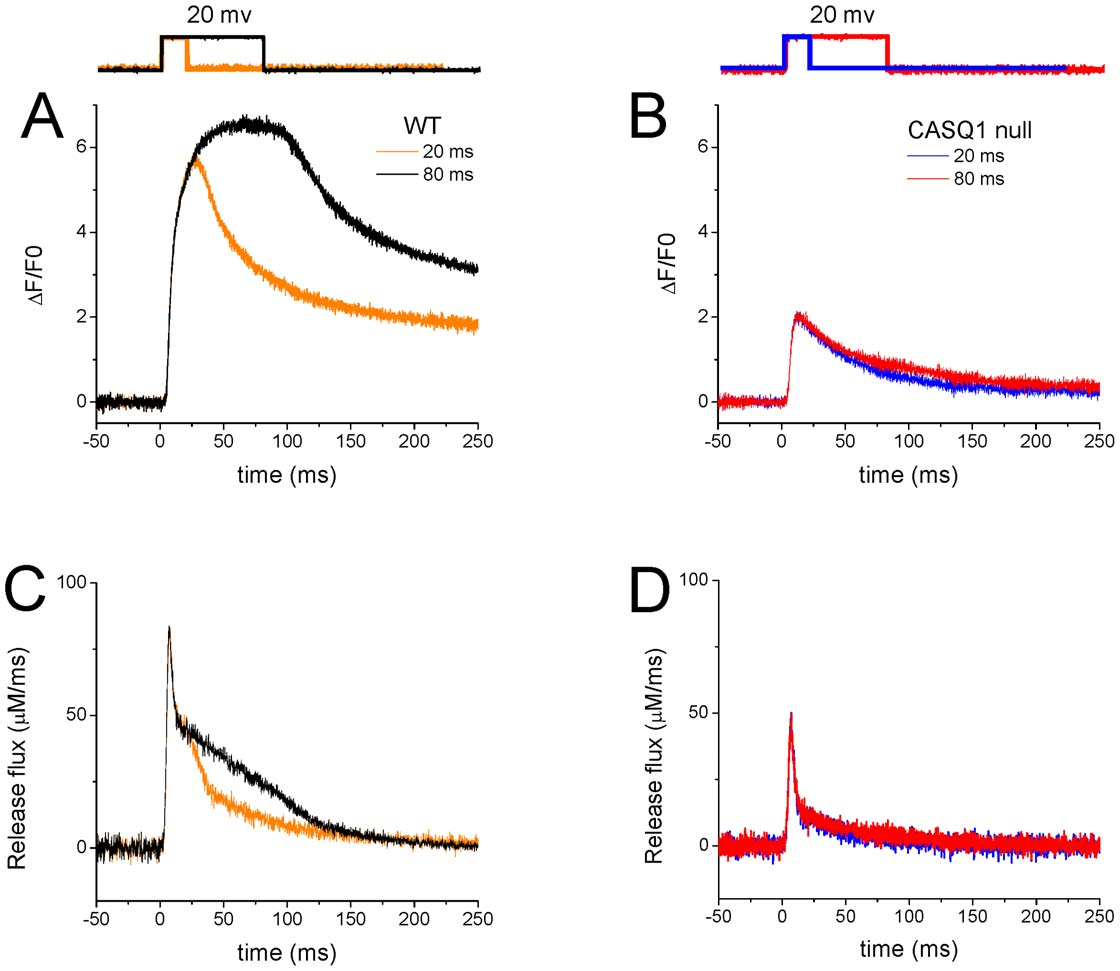
CASQ1 null fibers lack the slowly decaying component of release. Fluo-4 fluorescence transient measured as ΔF/F0 for WT (left) and CASQ1 null fibers (right). **A** is the time course of fluo-4 records averaged from 3 WT fibers and **B** is the time course obtained for CASQ1 null fibers averaged from 3 fibers. 20 mV depolarizing pulses were applied and subsequently terminated at either 20 ms or 80 ms as illustrated on top of figures **A** and **B** in order to evaluate the slowly decaying component of release in both WT and CASQ1 null fibers. **C** and **D** are Ca^2+^ release flux calculated from **A** and **B**. The results showed that CASQ1 buffering triggered a slowly decaying component of release in WT fibers while CASQ1 null fibers lacking this buffering does not exhibit this slowly decaying component of release.

In order to further explore the slowly decaying component of release in WT and CASQ1 null fibers, we terminated the pulse to +20 mV after either 20 or 80 ms. As shown in Fig. 9, terminating the pulse after 20 ms in WT fibers caused a clear decrease in the fluorescence signal (Fig. 9, A) and in the corresponding calculated Ca^2+^ release flux record (Fig. 8, C), indicating that release was still active when the pulse was terminated at 20 ms. Thus the slowly decaying component of release was clearly present in WT fibers since the rapidly decaying component was already terminated by 20 ms at +20 mV (Fig. 7H, inset). In contrast, in CASQ1 null fibers, terminating the pulse after 20 ms had essentially no effect on the either the fluorescence signal (Fig. 9 B) or on the calculated Ca^2+^ release flux (Fig. 9 D). Thus, Ca^2+^ buffering from CASQ1 appears to maintain SR Ca^2+^ levels so as to allow a slowly decaying component of release in WT fibers. In contrast, in CASQ1 null fibers this buffering is lacking and release ceases after the end of the rapidly decaying component. However, since neither the amplitude nor the decay time constant of the rapidly decaying component of release are altered in CASQ1 null fibers, one can conclude that CASQ1 buffering of the intra luminal SR Ca^2+^ plays no role in determining the rapidly decaying component of release.

### FDB myofibers from CASQ1 null mice have a decrease in releasable SR Ca^2+^ content

**Figure 10:**
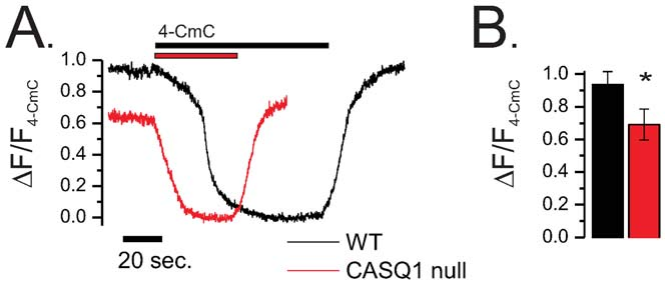
CASQ1 null myofibers have a reduction in relative SR [Ca^2+^]_free_. **A.** Representative SR Fluo5N fluorescence profile of a WT and CASQ1 null myofibers challenged with 1 mM 4-CmC. This profile was normalized to the maximum 4-CmC depletion (ΔF/F_4-CmC_). **B.** The evaluation of F_initial_ as a surrogate to SR [Ca^2+^]_free_ (see results) revealed that CASQ1 null myofibers had a ∼24% reduction in relative SR [Ca^2+^]_free_ compared to WT myofibers (Mann-Whitney rank-sum test, P<0.05).

A challenge with 1 mM 4-Chloro-m-Cresol (4-CmC) was used to assay maximal depletion of SR[Ca^2+^]_free_ in FDB myofibers SR loaded with the low affinity Ca^2+^ indicator Fluo5N,by a technique recently published by our group [42]. In both WT and CASQ1 null fibers, superfusion with 4-CmC initiated a rapid decrease to sustained nadir in intra-SR Fluo5N fluorescence that recovered with the removal of 4-CmC from the superfusate. This response is consistent with the evacuation and re-sequestration of Ca^2+^ by the SR. Fig. 10A displays representative normalized fluorescence profiles (i.e., ΔF/F_4-CmC_) of a WT and a CASQ1 null myofiber. The maximum sustained nadir in F_4-CmC_ was taken as the maximum depletion of SR Ca^2+^_free_ as 1 mM 4-CmC releases >98% of the releasable SR Ca^2+^ content [42]. With the nadir of the ΔF/F_4-CmC_ normalized to maximum SR depletion (e.g., zero SR Ca^2+^ content), the change in fluorescence (ΔF = F_initial_ - F_depetion_) was normalized to F_depetion_ to yield the ΔF/F_4-CmC_. In this presentation the ΔF/F_4-CmC_ at the start of the experiment minus the ΔF/F_4-CmC_ at the nadir represents the relative SR[Ca^2+^] content. Here we report that when compared to WT, CASQ1 null fibers exhibited a ∼24% reduction SR [Ca^2+^] compared to WT myofibers (Fig. 10B).

Additional qualitative insight was gained by examining the temporal evolution of the fluorescence during the 4-CmC challenge. We found that WT myofibers exhibited latency prior to the start of depletion compared to the CASQ1 null myofibers, in which SR Ca^2+^ content declined immediately. As these experiments were performed with bath perfusion of 4-CmC, quantitative analysis was not possible. However, we speculate that the absence of CASQ1 as a Ca^2+^ buffer was responsible for the immediate decline in fluorescence caused by agonist challenge in contrast to the slower decline in the WT fibers.

**Figure 11:**
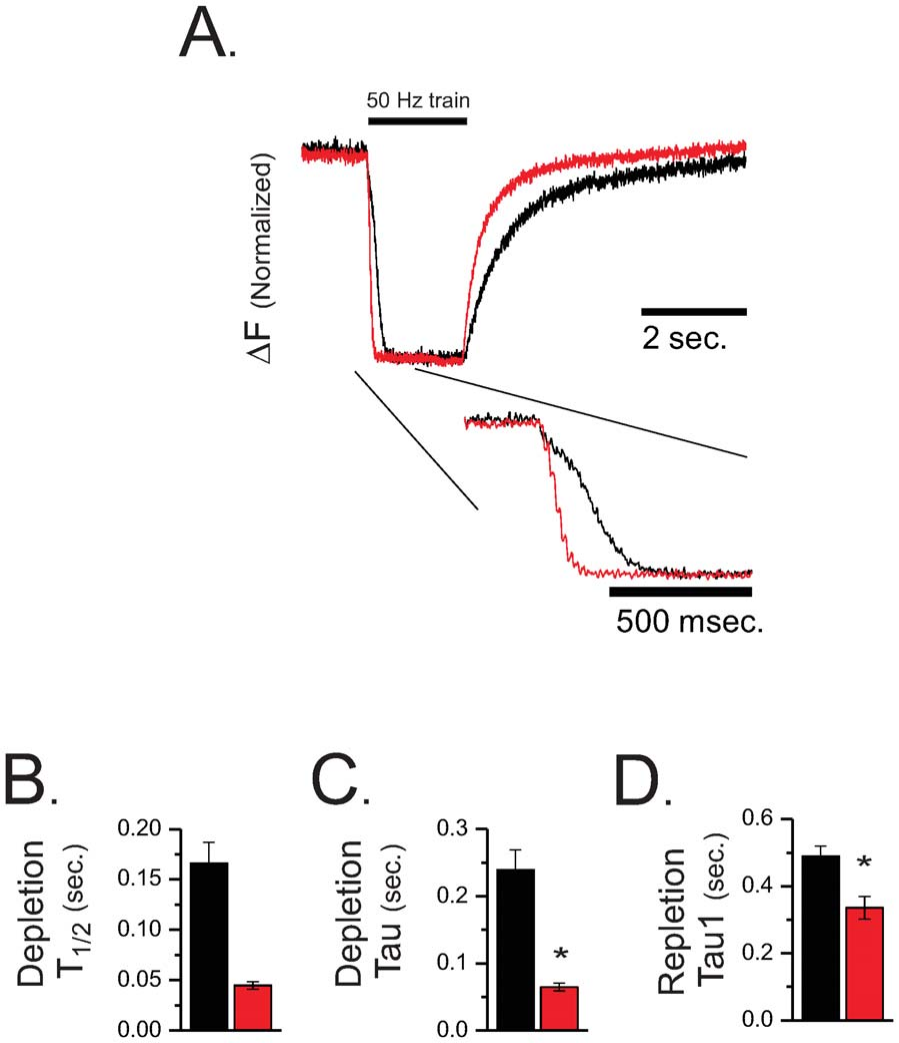
The dynamics of SR depletion and recovery are altered in CASQ1 null myofibers. **A.** Representative ΔF/F Fluo5N fluorescence profiles (normalized to max depletion) during 50 Hz sustained train of pulses. Offset lower panel is a time expanded trace. **B.** The half time of decay of the Fluo5N fluorescence signal during the 50 Hz train of stimulation was greatly reduced in the CASQ1 null fibers compared to the WT (0.046±0.004 sec. vs. 0.164±0.027, t-test, p<0.05) **C.** The evacuation rate of the SR was estimated by fitting the rapid fluorescence decline with a mono exponential function revealed a significantly faster evacuation rate (i.e., depletion tau), in CASQ1 null myofibers compared to WT (tau = 0.064±0.005 vs. 0.23±0.030 sec. respectively). **D.** The recovery of Fluo5N fluorescence following stimulation was best fit with a mono-exponential function in the WT and a two component exponential function in the CASQ1 null. A comparison of repletion Tau for the WT and the Tau of the initial exponential rise (Tau1) of the CASQ1 null revealed that CASQ1 null myofibers recovered more rapidly the WT myofibers (Tau1 = 0.323±0.034 vs. Tau = 0.488±0.031 sec. respectively, t-test, p<0.05).

A detailed evaluation of the temporal evolution of the SR[Ca^2+^] evacuation and recovery was assayed with prolonged 50 Hz trains of AP's using field stimulation. Recently we demonstrated that this protocol could produce ∼98% depletion of the SR[Ca^2+^]_free_ in WT myofibers [42]. Fig. 9A displays a representative ΔF/F Fluo5N fluorescence profile (i.e., F-F_stim_/F_stim_) normalized to the maximal depletion to highlight the temporal kinetics. Following the initiation of tetanic stimulation (0.5 ms pulse @ 50 Hz) in both genotypes, SR Ca^2+^ content reached the nadir relatively rapidly, and recovered soon after the stimulation was terminated. On an expanded time scale it was also evident that there was a delay prior to the final exponential decay of SR Ca^2+^ content in WT fibers that was not present in the CASQ1 null fibers. As a result, the decay time to the nadir was very much shorter in the CASQ1 null fibers (Fig. 11B). The evacuation rate of the SR, estimated by fitting the rapid fluorescence decline with a mono exponential decay, revealed a significantly faster evacuation rate in CASQ1 null myofibers compared to WT with the rate of depletion (Fig. 11C) of SR Ca^2+^ content was significantly faster in the CASQ1 nulls. This relationship can be better appreciated in the time expanded lower inset of panel 11A. Here the slight delay for WT myofibers to reach the maximal release rate is evident while the CASQ1 null myofibers drop rapidly from the start of the depolarization. This result in the CASQ1 nulls is consistent with the lack of an SR Ca^2+^ buffer to sustain SR [Ca_2+_]_free_ during release.

Finally, the recovery kinetics following stimulation were examined and taken as SERCA refilling the SR. This recovery of fluorescence was best fit with a mono-exponential function in WT fibers and a two component exponential function in the CASQ1 nulls, a finding consistent with the recent report by Canato et al [28]. A comparison of the time constant (Tau) for the WT and the Tau for the initial rise (Tau1) of the CASQ1 null revealed that CASQ1 null myofibers recovered more rapidly than WT myofibers (Fig. 9D; Tau1 = 0.323±0.034 vs. Tau = 0.488±0.031 sec. respectively).

## Discussion

Recent studies in muscle fibers with varied levels of CASQ1 [43] as well is in muscle fibers genetically depleted of CASQ1 and or CASQ1 and 2 [24], [27], [28], [44] have demonstrated that CASQ is not required for EC coupling, but have reaffirmed its importance as a Ca^2+^ buffer in skeletal muscle SR. However, in light of the importance of the identified changes in SR function in the absence of CASQ1, given that only minor changes in the functional contractility phenotype have been identified [24] despite severe dysfunction and death when environmental heat stress is imposed or after exposure to volatile anesthetics [44] there is an apparent incongruence between the functional phenotypes in relation to SR function.

In the present investigation we used assays of functional performance *in vivo* as well as assays of contractility and SR Ca^2+^ handling *in vitro* to gain additional insight into the functional role for CASQ1 in skeletal muscle. In summary, our main findings are: (A.) Both voluntary and involuntary exercise capacity was greatly reduced in the CASQ1 null mice. (B.) While a substantial deficit in peak force produced by the CASQ1 null EDL muscles could be almost fully accounted for by a decrease in mass these muscles exhibited a dramatic frequency dependent inability to sustain tension during a pulse train. (C.) With electric field stimulation of intact FDB fibers there is a significant decrease in the Ca^2+^ transients in CASQ1 null myofibers for single action potentials, and a collapse of the Ca^2+^ transient with repetitive trains of pulses. (D) Under voltage clamp, the peak SR Ca^2+^ release flux, as well as total SR Ca^2+^ release is significantly reduced in CASQ1 null fibers. The suppression of Ca2+ release flux in CASQ1 null fibers appears to be due to a selective suppression of the slowly decaying component of SR Ca^2+^ release (E.) There is a significant decrease in the SR [Ca^2+^]_free_ and a increase in the SR [Ca^2+^] decay and reuptake kinetics in CASQ1 null muscles consistent with a loss of an intra-SR Ca^2+^ buffering capacity. Taken together we have determined that the genetic ablation of CASQ1 expression results in significant deficits in contractile activation consistent with alterations in SR Ca^2+^ release and/or SR [Ca^2+^].

### The slowly decaying component of SR Ca^2+^ release is selectively eliminated in CASQ1 null fibers

An interesting novel result in the present report is the finding that the decrease in peak Ca^2+^ release flux in CASQ1 null fibers appears to be solely due to elimination of the slowly decaying component of SR Ca^2+^ release, whereas neither the amplitude nor the time course of the rapidly decaying component of SR Ca^2+^ release is altered. One possible explanation for this finding could be that the rapidly decaying component of release comes from an intra-SR Ca^2+^ pool that is not buffered by CASQ1, whereas the slowly decaying component comes from another SR pool that is buffered by CASQ1. Alternatively, the rapidly decaying component of release could already be completed by an early time point after the start of depolarization at which the intra-SR[Ca^2+^] had not yet significantly declined (e.g., earlier than 20 ms after the start of a pulse to +20 mV; see Fig. 9). Independent of the underlying mechanism, our findings support the general conclusion that CASQ1 null myofibers exhibit a temporal collapse in the Ca^2+^ transient due to a progressive failure to maintain the level of SR[Ca^2+^]_free_. The net result is that CASQ1 null myofibers show a significant decrease in the magnitude of peak SR Ca^2+^ release flux (∼55%) with no significant change in the voltage-dependence of Ca^2+^ release (Fig. 6A and B).

A recent paper has also focused on differences in Ca^2+^ release in voltage clamped WT or CASQ1 null FDB fibers using the same CASQ1 mutant mouse strain and very similar voltage clamp and Ca^2+^ indicator methods as used here together with simultaneous optical monitoring of SR luminal [Ca^2+^] [41], which was not done here. Based on their results using longer pulses than used here they concluded that SR Ca^2+^ release permeability (i.e., the ratio of SR Ca^2+^ release flux to SR[Ca^2+^]) is appreciably decreased 400 to 500 msec after the start of large depolarizing pulses in WT fibers. By contrast, our voltage clamp studies focused on events occurring during the first 10 to 20 msec following the start of similar large depolarizing steps. Independent of any possible later SR Ca^2+^ permeability changes in the WT fibers, our results showed that both amplitude and time course of the rapidly decaying component of SR Ca^2+^ release was essentially unchanged in CASQ1 null fibers, whereas the slowly decaying component of release was fully eliminated. Our use of relatively brief (20 ms) depolarizing pulses served as an empirical test of this interpretation. The late occurring events (i.e., >400 msec) studied by Sztretye et al. [41] by which SR Ca^2+^ permeability changed in WT fibers but not in CASQ1 null fibers, were not examined here. In contrast, by focusing on the events early during depolarization we show that the component of release that turns off within 10–20 ms of the start of a large depolarization is independent of the presence or absence of CASQ1, whereas the more slowly decaying component of release appears to be totally dependent on the presence of CASQ1.

A corollary to our conclusion that the slowly decaying component of SR Ca^2+^ release is eliminated in CASQ1 null fibers, but the rapidly decaying component is unchanged is that the total Ca^2+^ release from the SR should be considerably smaller in CASQ1 null than in WT fibers. Whether the 40% suppression in measured total SR Ca^2+^ released in EGTA buffered CASQ1 null fibers [41] corresponds to this prediction remains to be determined.

### CASQ1 null FDB myofibers have a decrease in the releasable SR[Ca^2+^]_free_

Based on recent reports with sustained or repetitive depolarizations, CASQ1 null myofibers are reported to have a reduction in the releasable SR [Ca^2+^]_free_ of between 20% [27] and >65% [28] when compared to WT. Here we report a ∼24% decrease in 4-CmC releasable SR [Ca^2+^]_free_ in CASQ1 null myofibers. In these experiments we used 5 mM BAPTA-AM to assist in the suppressing of movement during 4-CmC induced Ca^2+^ release (see [42]). As BAPTA at this concentration is likely to reduce Ca^2+^ dependent inactivation of the RyR1[41], [42], we believe that activation with 4-CmC (1 mM) represented maximal SR [Ca^2+^]_free_ depletion and is thus an accurate estimate of SR [Ca^2+^]_free_.

In the 4-CmC experiments, we observed an apparent increase in the rapid phase of SR depletion followed by an increase in the rate of SR[Ca^2+^] recovery. Quantification of these responses was not attempted however as the temporal initiation and cessation of the 4-CmC was in question with the bath superfusion. In experiments with prolonged trains of 50 Hz tetani we were able to precisely identify the temporal point of activation and termination and here we report a significant increase in the evacuation rate of SR[Ca^2+^]_free_ as well as an increase in rate of Ca^2+^ re-sequestration in the CASQ1 null fibers; results consistent with a decrease in the amount of intra SR buffer due to the absence of CASQ1. In WT muscle fibers, the CASQ1-Ca^2+^ store acts to sustain SR Ca^2+^ content, and thereby Ca^2+^ release. In the CASQ1 nulls this buffered store is absent, causing the Ca^2+^_free_ to be more rapidly depleted during activation. During re-sequestration, the CASQ1 in the WT muscle acts to sequester SR Ca^2+^ during refilling thus reducing the rate of the accumulation of SR[Ca^2+^]_free_.

### The decrease in *in vivo* and *in vitro* functional performance in CASQ1 null animals is consistent with a decrease in SR Ca^2+^ release

Based on the severe deficits in SR Ca^2+^ handling that have been reported in CASQ1 null fibers during prolonged depolarization under voltage clamp [27] as well as with more physiologically relevant trains of brief depolarizations [27], [28] we reasoned that functional phenotypes that relied on similar prolonged fiber activations would reveal concomitant loss-of-function phenotypes *in vivo* and *in vitro*.

To address muscle specific functional performance we performed contractility assays *in vitro* in EDL muscles. Consistent with previous reports by Paolini et al (2009) we identified a significant reduction of maximal twitch or tetanic force in the CASQ1 null (Fig. 2A). Interestingly, this deficit could be almost completely explained by the loss in muscle mass in the CASQ1 null animals rather than by the lack of CASQ1 per se. However, despite the similarity in force generation per unit muscle area, we demonstrate for the first time that CASQ1 null EDL muscle showed a frequency dependent collapse in the force transient (Fig. 3). These data are most consistent with decreased SR Ca^2+^ release/increased evacuability and/or reduction in Ca^2+^ store content that we and others [24], [27], [28], [41] have now shown in the CASQ1 null. An additional functional correlate of the SR Ca^2+^ handing phenotype that we observed in the CASQ1 null were the significant increases in the rate of rise and concomitant decrease in time to peak in force transients, which is consistent with the increased rate of intra-SR Ca^2+^ evacuation and the decreased time of Ca^2+^ re-sequestration relaxation we identified in these muscles (Table 1).

In the context of the *in vitro* functional phenotype we can consider more readily the functional phenotypes identified *in vivo*. Our assays identified a peripheral locomotor muscle specific functional deficit in the CASQ1 null mice. First, grip strength was markedly reduced and grip fatigue was increased in CASQ1 null mice; two non-specific *in vivo* assays of peripheral neuromuscular function. Second, we demonstrated that both voluntary and involuntary exercise capacity was greatly reduced in the CASQ1 null mice. It is important to note that in neither instance did the mice exhibit signs of distress or death during or following activity which would have indicated severe metabolic complications such as was demonstrated in these mice when challenged by environmental heat stress [44]. Furthermore, because CASQ1 is only expressed in the skeletal muscle and not in the heart, this loss-of-function exercise phenotype is likely independent of cardiovascular function deficits identified in the CASQ2 null mice [25]. Instead we believe functional performance was limited by intrinsic deficits in skeletal muscle performance (i.e., peripheral locomotor and/or respiratory) due to the lack of CASQ1.

It is clear that *in vivo* physiologic function was likely affected by a complex array of deficits including the loss in muscle mass and deficits in SR Ca^2+^ handling identified here. The decrease in exercise capacity and increased fatigability is in apparent contrast however to the report that EDL muscles from CASQ1 null mice fatigue slower *in vitro* than WT [24]. The decrease in EDL fatigue in CASQ1 null mice *in vitro*, which we have quantitatively confirmed (data not shown), has been recently addressed by Zhao et al. [8] who demonstrate that CASQ1 knockdown with siRNA results in a significant increase in store operated Ca^2+^ influx (SOCE) in apparent compensation for the increased depletion of the SR during muscle fiber activation. Based on our functional assessments *in vivo* (i.e., treadmill, running wheel, grip) it is clear that this enhancement in SOCE that decreases fatigue *in vitro* does not fully compensate for the EC coupling dependent processes that are needed to sustain physiologic function *in vivo*.

## Methods

### Animal Models

All experiments involving animals were conducted according to the National Institutes of Health *Guide for the Care and Use of Laboratory Animals*, and were approved by the Institutional Animal Care and Use Committee of the University Of Maryland School Of Medicine (Approval # 1210009). C57BL/6 CASQ1 null mice [24] were raised at Charles River Laboratories, and shipped to the animal facility at 4–8 wks of age. All experiments were conducted on adult mice (3 month±10 days). Aged matched C57BL/6 mice, obtained from Charles River laboratories, were used as controls.

### Gross neuromuscular performance

*Voluntary exercise capacity* was assessed in previously sedentary CASQ1 null (n = 6) and wild type (WT; n = 6). Mice were singly housed in a cage with a mouse running wheel (Lafayette Instruments, Lafayette, IN) in a 12/12-hour light/dark animal facility with *ad libitum* access to food and water. The number of wheel revolutions was computer analyzed per 24-hour period and data was converted to mean number of meters per day and reported as descriptive data.

*Treadmill exercise capacity* (i.e., non-voluntary) was evaluated in previously sedentary CASQ1 null (n = 6) and WT (n = 6). The protocol was similar that of Massett and Berk [45] except with slight modifications. On day 1 the mice were acclimatized to the treadmill compartment for 5 min without treadmill activity. On days 2 and 3 mice were acclimated to the treadmill at a low speed (0% grade; 10 & 12 m/min respectively). On Day 4 mice were exercised at 10 meters/minute for the first 10 min then ramped to 20 m/min over 1 min and held there until volitional exhaustion. Prior to fatigue mice were encouraged to maintain running pace by non-noxious “startle”; a tap on the hindquarter with an 11 inch forceps. Volitional exhaustion was assessed by a single rater, blinded to mouse genotype, who determined the time point at which the mouse was unable to maintain treadmill pace after two successive startles. After the trial, total time was converted to distance covered. Differences in total distance by group were analyzed by Student's t-test.

*Forepaw grip strength* was determined on a commercial rodent grip strength meter (Columbus Inst., USA). Peak grip strength was assessed in 7 successive trials separated by 10–15 seconds by a single rater blinded to genotype. The highest two values were averaged, normalized to bodyweight and used as a measure of the peak grip strength. The decrement in force between the peak value and the value of the 7^th^ trial was evaluated as an index of fatigue.

### *In vitro* muscle contractility

Muscle performance of the *extensor digitorum longus* (EDL) was assessed *in vitro* using methods described previously [34]. In brief, single EDL muscles were surgically excised with ligatures at each tendon (5-0 silk suture) and mounted in an *in vitro* bath between a fixed post and force transducer (Aurora 300B-LR) operated in isometric mode. The muscle was maintained in physiological saline solution (PSS; pH 7.6) containing (in mM) 119 NaCl, 5 KCl, 1 MgSO_4_, 5 NaHCO_3_, 1.25 CaCl_2_, 1 KH_2_PO_4_, 10, HEPES, 10 glucose, and maintained at 30°C under aeration with 95 O_2_/5 CO_2_ (%) throughout the experiment. During a 5 min equilibration, single twitches were elicited at every 30 seconds with supra-maximal electrical pulses (200 µsec.sec) via platinum electrodes running parallel to the muscle. For brief tetanic stimuli 250 msec trains of pulses were delivered at 1, 10, 20, 40, 60, 80, 100, 150 Hz and isometric tension was evaluated. After the experimental protocol, the muscle rested for 5 min at which time muscle length was determined with a digital micrometer, and the muscle was trimmed proximal to the suture connections, blotted and weighed. The cross-sectional area for each muscle was determined by dividing the mass of the muscle (g) by the product of its length (*L*_o_, mm) and the density of muscle (1.06 g/cm^3^; [36]). Muscle performance is expressed as isometric tension (g.mm^2^) per cross-sectional area. Statistical comparisons between groups were performed using ANOVA procedures (SigmaStat v3.1). Significance set at p<0.05.

### FDB fiber preparation

Fibers were prepared using enzymatic dissociation of flexor digitorum brevis (FDB) muscles of 6- to 7-week-old CASQ1 null and WT mice, and were cultured as previously described [46].

### Indo1 ratiometric recordings

Indo1 AM ratiometric recording and analysis were performed as previously described [47] but with some modifications for loading. Briefly Cultured FDB fibers were loaded with indo1-AM (2 µM for 30 min at 22°C; Invitrogen, Eugene, OR) in L-15 media (ionic composition in mM: 137 NaCl, 5.7 KCl, 1.26 CaCl2, 1.8 MgCl2, pH 7.4; Invitrogen, Eugene, OR). Then the fibers were washed thoroughly with appropriate L-15 media to remove residual Indo1 AM and incubated at 22°C for another 30 min to allow dye conversion. The culture dish was mounted on an Olympus IX71 inverted microscope and viewed with an Olympus 60×/1.20 NA water immersion objective. Fibers were illuminated at 360 nm, and the fluorescence emitted at 405/30 and 485/25 nm was detected simultaneously. The emission signals were digitized and sampled at 2 KHz using a built-in AD/DA converter of a EPC10 amplifier and the acquisition software Patchmaster (HEKA, Instruments). Field stimulation (1 ms, 8–14 V and alternating polarity) was provided by a custom pulse generator through a pair of closely spaced (0.5 mm) platinum electrodes.

### Electrophysiology

Membrane current measurements were performed using the whole-cell configuration of the patch-clamp technique [48] with modifications for mammalian FDB fibers detailed previously. [38], [49], [50]. Whole cell Ca^2+^ current measurements were carried out using external solution containing the following (in mM): 150 TEA-CH3SO3, 2 CaCl2, 1 MgCl2, 10 Hepes, 0.001 TTX, 1 4-aminopyridine, 0.025 N-benzyl-p-toluene sulfonamide (BTS), and pH of solution adjusted to 7.4 with CsOH. The pipette solution (internal) contained (in mM): 140 Cs-CH3SO3, 10 Hepes, 20 EGTA, 6 MgCl2, 11.5 CaCl2, 4 Na2ATP, 14 creatine phosphate, 0.3 Na2GTP, and 0.1 leupeptin and solution pH adjusted to 7.4 with CsOH to obtain a buffered solution.

### Fluo-4 high-speed line-scan (x-t) confocal microscopy

Fluo-4 fluorescence line-scan (100 µs/line) confocal microscopy measurements were carried out on a Zeiss LSM 5 Live system as previously described [38] Fibers were dialyzed for 20 minutes via the patch pipette with 50–75 µM fluo-4 5K^+^. Typically, the voltage depolarization steps were applied 300 ms after the start of the confocal scan sequence, thus providing control images prior to stimulation at the start of each sequence. These control images were used to determine the resting steady-state fluorescence level (F_0_). Average intensity of fluorescence as a function of time (F(x,t)) within selected areas of interest (AOIs) were measured using Image Examiner (Carl Zeiss, Jena). The average F_0_ value in each AOI prior to test pulse is used to scale fluo4 fluorescence in the same AOI as F/F_0_.

### Time course of free myoplasmic Ca^2+^ concentration

In order to estimate the time course of free [Ca^2+^] during a Ca^2+^ transient in these fibers the fluorescence F/F0 signal must be corrected for both dye saturation and for the kinetic delay in dye equilibrium. The kinetic lag and dye saturation can be corrected using an approach previously described for Fura-2 ratio signals [51] but adapted here for F/F0 records [52]. Under conditions when the dye is not at equilibrium with the cytosolic free Ca^2+^, the time course of cytosolic free Ca^2+^concentration ([Ca]cyto(t)), is given by:
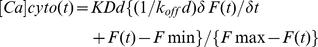
(1)(see [51]), Eq. 5, with rearrangements), where KDd is the dissociation constant for Ca^2+^ from the indicator dye, koffd is the off rate constant for Ca^2+^ dissociation from the dye, F is the fiber fluo-4 fluorescence signal and Fmax and Fmin are the fluorescence at saturating and zero free Ca^2+^, respectively.

When the concentration is changing slowly, the following condition applies:
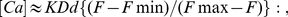
(2)Then, average [Ca^2+^]cyt (t) is derived from F(t)/F0 [52] as:
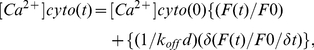
(3)where [Ca^2+^]cyto(0) is free cytosolic [Ca^2+^] at resting levels. This equation assumes that the dye is far from saturation by Ca^2+^, which appears justified for fluo-4 considering that [Ca^2+^]cyto was always much lower than the value of fluo-4's KD previously determined using in situ calibrations [38]. [Ca^2+^]cyto(0) was set to 0.081 µm. This assumption was roughly consistent with measurements of resting [Ca^2+^] under similar experimental conditions using the ratiometric indicator indo-1 [53]. The indo-1 fluorescence emission ratio did not change significantly with time starting 20 min after whole-cell configuration establishment.

### Calculation of Ca^2+^ release flux

Under the conditions of 20 mM EGTA with added Ca^2+^ in the pipette, EGTA still the major binding site for released Ca^2+^. In that case d [Ca-EGTA](t)/dt provides a good approximation to the rate of SR Ca release in the fiber, and will be used in the Ca^2+^ release calculations presented here. For Ca^2+^ binding to EGTA,
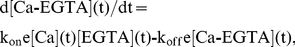
(4)where k_on_e and k_off_e are on and off rate constants for Ca^2+^ binding to EGTA.
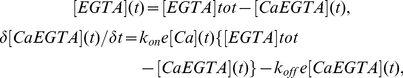
(5)Equation 5 was solved numerically using [Ca^2+^]cyto(t) from Eq. 3, and using the Euler method to calculate the time course of d[Ca-EGTA](t)/dt, which was used as the time course of the rate of Ca^2+^ release from the SR during the voltage clamp pulses. [EGTA] and [Ca-EGTA] inside the fiber at the start of each pulse were assumed to be 70% of that estimated for the pipette solution (obtained with the programs Maxchelator [54]. and Bound and determined [55]. The parameter values used in our calculations were k_off_d = 90 s-1 [52], [56] and k_off_e = 1.2 s-1 (EGTA *KD* = 0.08 µM).

### Assay of free intra-SR [Ca^2+^]

Methods for the Fluo5N loading of FDB's and measures of 4-CmC and electrical depletion were conducted as previously described [42]. In brief, enzymatically isolated FDB's were loaded in a solution of fluo-5N (Invitrogen, Carlsbad, CA) dissolved in 20% pluronic in DMSO, and diluted in DMEM to a final concentration of 10 µM. Loading was at 37°C for 2 hr, to allow uptake of dye into the cytosol and its de-esterification in the lumen of the SR. Following loading, cells were incubated for 1 hr at room temperature followed by 1 hr at room temperature in medium containing 5 µM 1,2-Bis(2-aminophenoxy)ethane-N,N,N′,N′-tetraacetic acid tetrakisacetoxymethyl ester (BAPTA-AM;Invitrogen, Carlsbad, CA) dissolved in 20% pluronic in DMSO) and 25 µM *N*-benzyl-*p*-toluene sulfonamide (BTS; Sigma Aldrich, St. Louis, MO; dissolved in DMSO), to prevent contraction during imaging. During 37°C loading of fluo-5N, fibers were plated on cover slips coated with Matrigel (BD Biosciences, San Jose, CA). BAPTA-AM also reduced the fluorescence arising from fluo-5N molecules remaining in the cytosol and minimized the loss of fibers caused by the maximal release of Ca^2+^ from the SR. Fibers were studied within 2 hr after loading was terminated.

Fibers loaded with fluo-5N were imaged in normal Tyrode's solution (NT), containing (in mM), 140 NaCl, 0.5 MgCl_2_, 0.3 NaH_2_PO_4_, 5 HEPES, 5.5 glucose, 1.8 CaCl_2_, and 5 KCl, pH of7.4 with NaOH. Confocal images were acquired on an inverted microscope (Axiovert 200 M LSM-510; Carl Zeiss, Germany) equipped with an oil immersion objective (40×, 1.2 NA) and a 488 nm excitation laser in line scan (*xt*) mode.

Changes in the fluo-5N signal in response to application of 1 mM 4-chloro-m-cresol (4-CmC) in the bath were recorded with the confocal microscope in line scan mode. Fluo-5N was excited with a 488 nm laser and emitted light was filtered through a long pass, 505 nm filter.

Electrical stimulation (0.5 ms pulse duration, 40 V/cm^2^, 1–50 Hz for 10 s) was used to trigger incremental SR Ca^2+^ release. Stimulation was produced with a Myopacer (IonOptix, Milton, MA) through platinum electrodes placed on either side of the fiber of interest. Following a brief period of inactivity, we rapidly perfused 4-CmC onto the fiber (see above) to measure its maximal release of Ca^2+^ from the SR. Stimulation elicited SR release was then normalized to the 4-CmC result.

### Statistical analysis

Statistical analyses were performed using OriginPro 8.0 or SigmaStat 3.5. All data are presented as mean ± S.EM. unless otherwise noted. Statistical significance was assessed using either parametric two sample t-test, ANOVA or with the non-parametric Mann-Whitney rank-sum test as indicated. Significance was set at P<0.05.
